# Genetic and Epidemiological Aspects of Louis-Bar Syndrome Transmission: The Impact of Consanguineous Marriages on the Incidence of Hereditary Disorders

**DOI:** 10.34763/jmotherandchild.20252901.d-25-00038

**Published:** 2025-12-24

**Authors:** Zhanyl Baitokova, Nursultan Erkinbek uulu, Ajgul Matkeeva, Maral Turdumatova, Askarbekova Zhyldyz

**Affiliations:** Department of Public Health International Higher School of Medicine (IHSM) 720054, 1 F Intergelpo Str., Bishkek, Kyrgyz Republic; National Center of Maternity and Childhood Care 720038, 190 Akhunbaev Str., Bishkek, Kyrgyz Republic; Department of Neurology and Clinical Genetics named after A.M. Murzaliev I.K. Akhunbaev Kyrgyz State Medical Academy 720020, 92 Akhunbaev Str., Bishkek, Kyrgyz Republic; Department of Faculty Pediatrics I.K. Akhunbaev Kyrgyz State Medical Academy 720020, 92 Akhunbaev Str., Bishkek, Kyrgyz Republic; First Children’s Hospice 720016, 299/5 Chingiz Aitmatov Ave., Bishkek, Kyrgyz Republic

**Keywords:** Mutations, Autosomal Recessive Inheritance, Immunodeficiency, Cerebellar Atrophy, Family Genetic Counselling, Telangiectasia, Paediatrics, Paediatric Neurology, MRI Diagnostics, Differential Diagnostics, Ataxic Form of Cerebral Palsy, Palliative Care

## Abstract

**Background:**

The aim of this study was to investigate the genetic and epidemiological aspects of Louis-Bar syndrome transmission in the population of Kyrgyzstan, with a particular focus on the impact of consanguineous marriages.

**Methods:**

The study presents a clinical case of a family with three children affected by this disorder. All children exhibited characteristic manifestations, including progressive cerebellar ataxia of varying severity; conjunctival and cutaneous telangiectasias; recurrent infections; and delayed psychomotor development. In the eldest child, the clinical presentation resembled the ataxic form of cerebral palsy. Standardised scales assessing motor, manual, and communicative functions were used to evaluate the severity of ataxia.

**Results:**

Brain magnetic resonance imaging confirmed cerebellar atrophy in the eldest child and cerebellar subatrophy in the middle and youngest children. All children demonstrated telangiectasias on the mucous membranes of the eyes and skin, as well as signs of immunodeficiency manifesting as frequent infections. Family pedigree analysis revealed consanguinity in the third generation (the maternal grandmother and paternal grandfather were biological siblings). Molecular genetic testing identified a homozygous c.5932G > A mutation in the ATM gene encoding a protein involved in DNA repair.

**Conclusion:**

The findings confirm that consanguineous unions increase the risk of developing Louis-Bar syndrome, as they elevate the likelihood of inheriting identical mutant alleles. This study highlights the importance of medical-genetic counselling and prenatal diagnostics in families at high risk of hereditary diseases, particularly in regions with a high prevalence of consanguineous marriages.

## Introduction

Louis-Bar syndrome (LBS), also known as ataxia-telangiectasia (A-T), is a rare inherited disorder that leads to severe neurological impairment, immunodeficiency, and a substantial decline in patients’ quality of life. Its diagnosis presents a significant challenge due to the heterogeneity of its clinical manifestations, and the risk of inheritance is elevated in populations with a high frequency of consanguineous marriages. This renders the study of the genetic and epidemiological dimensions of the syndrome particularly pertinent. The condition is characterised by a combination of progressive cerebellar ataxia, telangiectasias, and immunodeficiency [1]. Patients often exhibit coordination deficits stemming from the disease’s cerebellar involvement, which manifest in early childhood as gait instability, impaired fine motor skills, and, in later stages, dysarthria and dysphagia. Telangiectasias – visually resembling small vascular “spider veins” – are localised to the skin and mucous membranes, particularly the sclerae and ear regions. Immunodeficiency results in heightened susceptibility to respiratory and middle ear infections, which exacerbate the disease’s course [2].

The underlying cause of Louis-Bar syndrome is mutations in the ATM (ataxia-telangiectasia mutated) gene, which is located on chromosome 11 [3]. This gene encodes the ATM kinase, which plays a critical role in DNA repair, cell cycle regulation, and apoptosis. Mutations in the ATM gene disrupt these essential processes, which increases cellular sensitivity to ionising radiation; impairs cell cycle regulation; and promotes the development of malignancies, particularly leukaemia and lymphoma [4]. A-T is inherited in an autosomal recessive manner, meaning a child must inherit mutant alleles from both parents to develop the disease. When both parents are carriers of a mutated ATM gene, the probability of having an affected child is 25% [5]. The syndrome typically follows a severe, progressive course, leading to early disability and reduced life expectancy. Multisystem involvement complicates diagnosis and treatment, as the disorder affects the nervous, immune, and vascular systems, resulting in a complex constellation of symptoms. Currently, there is no effective curative treatment, making genetic diagnostics and counselling pivotal tools for identifying at-risk populations and reducing disease incidence.

In populations with a high incidence of consanguineous marriage, the likelihood of disease inheritance increases due to the elevated risk of homozygosity for pathogenic mutations [6]. This is attributable to the increased probability that close relatives will carry the same genetic variants. Epidemiological studies estimate the prevalence of Louis-Bar syndrome to range from 1 in 40,000 to 1 in 100,000 live births, but in isolated populations with pronounced inbreeding, the frequency can be significantly higher [7]. In Kyrgyzstan, where consanguineous marriages are culturally prevalent, Louis-Bar syndrome represents a significant medical-genetic concern. The high rate of consanguinity in the country is driven by cultural traditions, religious beliefs, and geographic isolation in certain regions. Therefore, the study of the genetic and epidemiological factors of A-T in Kyrgyzstan holds substantial scientific and practical value.

Nissenkorn and Ben-Zeev [8] provide a clinical overview of A-T manifestations that encompasses their neurological, immunological, dermatological, and oncological dimensions. The authors discuss current approaches to diagnosis and treatment, as well as prospects for therapeutic innovation. They note the phenotypic heterogeneity of Louis-Bar syndrome, which is reflected in the broad spectrum of clinical symptoms and variability in age of onset. Disease severity may range from mild forms with late onset and slow progression to severe variants with early onset and rapid development of complications.

Amirifar et al. [9] also underscore the phenotypic variability of the disorder, emphasising that symptom severity depends on the nature of mutations in the gene responsible for DNA repair. The authors report heterozygous carriage rates for mutant alleles between 1.4% and 2%, and describe over 1,400 unique mutations, including null variants resulting in truncated proteins. Their study confirms that individuals with pathogenic mutations exhibit the classical clinical features of Louis-Bar syndrome. The article also analyses causes of death in A-T patients, highlighting the primary role of pulmonary failure in early childhood and malignancies in older children and adolescents. The authors also report the beneficial effects of modern treatment modalities (e.g., antibiotic therapy, immunoglobulin replacement) on patient longevity. Prognosis is closely linked to the severity of immunological deficits, which influence infection frequency, neurodegeneration, and cancer risk [9].

A comprehensive analysis of the genetic basis of Louis-Bar syndrome is presented by Rothblum-Oviatt et al. [10], who offer a detailed account of mutations in the ATM gene and their respective impacts on DNA repair, cell cycle control, and apoptotic mechanisms. The authors distinguish between two clinical forms, “classic” and “mild,” which differ in their clinical progression. The paper also discusses diagnostic challenges and advocates for a multidisciplinary treatment approach. A significant portion of the study is also dedicated to the pathophysiological mechanisms of the syndrome as it explores the consequences of impaired DNA repair on oncogenesis, immunodeficiency, and neurodegeneration. The review additionally evaluates current therapeutic strategies, including symptomatic treatment, immune modulation, and management of pulmonary infections, which, according to the authors, have contributed to increased life expectancy in affected individuals [10].

Existing scientific studies have insufficiently addressed the impact of consanguineous marriages on the genetic mechanisms underlying the transmission of ataxia-telangiectasia (A-T) in populations with a high level of inbreeding. The objective of the present study was to determine the influence of consanguinity on the development of A-T; to analyse the clinical manifestations of the disorder; and to identify characteristic mutations in the gene responsible for DNA repair. To achieve this, a comprehensive analysis was conducted – encompassing clinical presentation, assessment of cerebellar structural changes based on neuroimaging data, molecular genetic testing, and pedigree analysis – to identify hereditary risk factors.

## Literature review

The clinical presentation of A-T is diverse and affects multiple organ systems. The most characteristic features include impaired motor coordination (ataxia), difficulties in voluntary eye movement control (oculomotor apraxia), speech disturbances (dysarthria), involuntary movements (choreoathetosis), muscle rigidity (dystonia), and peripheral nerve dysfunction (peripheral neuropathy). Cognitive impairments, including difficulties in thinking, learning, and memory, are also commonly observed.

A visually distinctive sign is the presence of telangiectasias, which are dilated blood vessels most frequently visible on the sclera, facial skin, and neck.

Patients with A-T exhibit immunodeficiency, making them more susceptible to various infections, particularly of the respiratory tract. These may include frequent colds, sinusitis, otitis media, pneumonia, and viral infections.

Another significant concern is heightened sensitivity to ionising radiation, which increases the risk of malignancies, particularly leukaemias, lymphomas, and brain tumours.

Endocrine abnormalities may also be present, such as delayed puberty, diabetes mellitus, and thyroid dysfunction. Other potential manifestations include growth retardation, microcephaly, premature ageing, and a predisposition to autoimmune diseases.

The pathogenesis of A-T is primarily driven by mutations in the ATM gene, located on the long arm of chromosome 11 (11q22.3). This gene encodes the ATM protein, a large serine/threonine protein kinase belonging to the phosphatidylinositol 3-kinase-related kinase (PIKK) family. ATM is a key component of the genome stability maintenance system, mediating the cellular response to DNA damage.

Functions of the ATM protein:

1. DNA damage recognition. ATM is activated in response to various types of DNA damage, including double-strand breaks, single-strand breaks, oxidative damage, and chromatin structure abnormalities [11].
2. Signal transduction. Activated ATM phosphorylates a wide array of target proteins involved in cell cycle regulation, DNA repair, apoptosis, transcription, and other cellular processes [12].
3. Cell cycle control. ATM initiates a cascade of signalling pathways that arrest the cell cycle at the G1, S, and G2 phases, allowing time for DNA repair and preventing replication of damaged DNA [13].
4. Regulation of DNA repair. ATM plays a central role in coordinating multiple DNA repair pathways, including homologous recombination, non-homologous end joining, nucleotide excision repair, and mismatch repair [14].
5. Induction of apoptosis. When DNA repair is impossible or when extensive damage is present, ATM triggers apoptosis to prevent the accumulation of genomically unstable cells and reduce cancer risk [15].
6. Regulation of immune response. ATM is involved in the development and function of the immune system, including V(D)J recombination, lymphocyte maturation, and antigen response [16].
7. Maintenance of redox homeostasis. ATM modulates cellular responses to oxidative stress, protecting cells from damage caused by reactive oxygen species [17].

To date, the ClinVar database has registered over 4,000 mutations in the ATM gene that are associated with A-T. The majority of these are pathogenic mutations leading to loss of ATM protein function. The mutational spectrum of the ATM gene is highly heterogeneous and includes:

1. Missense mutations (approximately 60%), which result in amino acid substitutions in the ATM protein and impair its function [18];
2. Nonsense mutations (approximately 20%), which cause premature translation termination and the production of a truncated, non-functional protein [19];
3. Splice-site mutations (approximately 10%), which disrupt mRNA splicing and may lead to exon skipping, intron retention, or frameshifts [20];
4. Small deletions or insertions (approximately 5%), which involve the loss or insertion of a few nucleotides in the ATM gene and may result in frameshift mutations and impaired protein synthesis [21]; and
5. Large genomic rearrangements (approximately 5%), including deletions, duplications, and inversions affecting substantial portions of the ATM gene [22].

The risk of having a child with A-T in a consanguineous union depends on the degree of kinship and the carrier frequency of ATM gene mutations in the population. The closer the genetic relationship, the higher the risk. For instance, the risk is significantly greater for children born to first cousins than for those born to second cousins [23,24,25,26,27].

Mechanisms underlying increased risk in consanguineous marriages:

1. Increased inbreeding coefficient. Consanguineous unions elevate the inbreeding coefficient (F), increasing the probability that two alleles in an individual are identical by descent [28].
2. “Founder effect.” In isolated populations with a high prevalence of consanguinity, a “founder effect” may occur, whereby a single ATM gene mutation becomes widespread and accounts for the majority of A-T cases [29].

Consanguineous marriage is a complex social and cultural phenomenon shaped by numerous factors, including religious traditions, socio-economic conditions, geographic isolation, and ethnic customs. In some cultures, consanguineous unions are preferred, as they are believed to preserve family wealth, strengthen familial ties, and uphold cultural traditions [30]. It is crucial, however, to recognise that consanguinity is associated with an increased risk of genetic disorders, including A-T [31,32,33].

In conclusion, A-T is caused by mutations in a gene critical for DNA repair, leading to severe neurological and immunological consequences. The high frequency of consanguineous marriages increases the risk of homozygous inheritance of pathogenic mutations, necessitating the active implementation of molecular genetic diagnostics and preventive strategies. Genetic research, epidemiological surveillance, and educational programmes may contribute to reducing disease incidence and improving quality of life for affected individuals.

## Materials and methods

This study presents a retrospective analysis of a clinical case involving a family with three children affected by Louis-Bar syndrome. This approach enabled a detailed examination of the phenotypic variability of the disorder, its clinical manifestations, and the influence of genetic factors on the disease’s progression. The research was conducted in the Department of Neurology at the National Centre for Maternal and Child Health in Bishkek (Kyrgyzstan) from 2019 to 2021. This facility was selected because it specialises in the diagnosis and treatment of hereditary disorders of the nervous system and thus provided access to a wide range of diagnostic modalities.

The study included three children aged between two and eight years, all with confirmed diagnoses of Louis-Bar syndrome. The familial relationships of the patients indicated an autosomal recessive pattern of inheritance, underscoring the importance of pedigree analysis in identifying risk factors. This study also evaluated various diagnostic approaches, including clinical, instrumental, laboratory, and molecular genetic investigations.

Special emphasis was placed on the assessment of neurological impairments, motor skills, and cognitive functions. Standardised international classification systems were used for objective evaluation. The Gross Motor Function Classification System (GMFCS) was applied to determine the level of motor activity and the need for assistance with mobility. The Manual Ability Classification System (MACS) assessed hand function and degree of independence in daily activities. The Communication Function Classification System (CFCS) was utilised to evaluate communicative abilities, including the level of social interaction and degree of speech impairment. The use of these scales allowed for objective clinical data assessment to help identify correlations between the severity of cerebellar atrophy and the extent of motor and cognitive impairments.

Treatment modalities were analysed in terms of their effectiveness. Supportive therapy included the administration of antioxidants such as L-carnitine, coenzyme Q10, and vitamin E, alongside immunoglobulin replacement therapy to correct immunodeficiency. The efficacy of antibiotic therapy, bronchodilators, and respiratory therapy in reducing the incidence of infectious complications was also evaluated. A comparative analysis of clinical data revealed a relationship between the severity of cerebellar atrophy and disease severity and helped identify the most effective approaches to supportive care.

A comprehensive medical history was collected, including data on disease onset, symptom dynamics, comorbidities, family history, and psychomotor development. Neurological examination enabled the assessment of muscle tone, strength, reflexes, coordination, speech, and the identification of characteristic cerebellar signs, such as nystagmus, dysarthria, and dysmetria.

To assess patients’ cerebellar changes, brain MRI was performed using a Siemens Magnetom Aera 1.5 Tesla scanner (Germany). Additional electrophysiological tests were conducted, including electroneuromyography (ENMG) to diagnose peripheral neuropathy. Evoked potentials, including visual (VEP), somatosensory (SSEP), and motor (MEP), were employed to evaluate the functional integrity of patients’ central and peripheral sensory pathways, which proved essential for the differential diagnosis of ataxias.

Laboratory tests included immunological assessments. Quantification of immunoglobulin levels (IgA and IgG) and alpha-fetoprotein (AFP) allowed for the identification of immunodeficiency patterns characteristic of Louis-Bar syndrome. To better understand the genetic contribution, an extended pedigree was constructed that analysed hereditary links. Management strategies were comparatively evaluated based on the severity of the clinical manifestations and neuroimaging findings.

## Results

### Genetic mechanisms and clinical variability of Louis-Bar Syndrome

Louis-Bar syndrome is a hereditary disorder caused by mutations in the ATM gene, which plays a critical role in DNA repair and genomic stability maintenance [3]. Despite its clear genetic aetiology, clinical manifestations of Louis-Bar syndrome vary significantly, influenced not only by the type of mutation but also by epigenetic and population-based factors. The severity of the syndrome is associated with the specific nature of the ATM mutation, which determines residual protein activity and the extent of its functional disruption [12].

**Figure 1.**
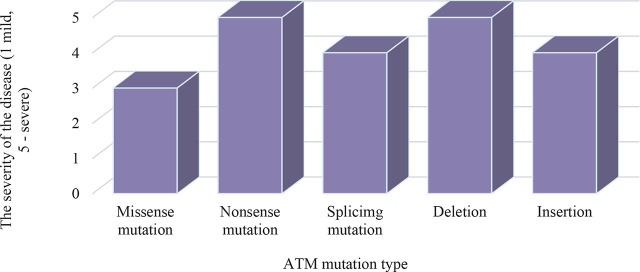
Impact of ATM mutation type on the severity of Louis-Bar Syndrome. *Source:* created based on analysis of data [13,14,15,16,17,18].

Missense mutations are the most commonly observed and may retain partial ATM protein activity, resulting in a milder clinical course [13]. In contrast, nonsense mutations and large deletions that lead to premature termination of protein synthesis are linked to the most severe phenotypes of the disease, which are characterised by early-onset ataxia, profound immunodeficiency, and a markedly increased risk of malignancies [14]. Splicing mutations and insertions have an intermediate level of impact depending on their precise genomic location and their effect on mRNA processing [15]. In populations with high levels of inbreeding, specific pathogenic ATM variants predominate due to the founder effect, increasing the likelihood of homozygous mutation carriage [16] (Figure 1).

The diagram illustrates that nonsense mutations and large deletions result in the most severe forms of Louis-Bar syndrome due to the complete loss of functional ATM protein synthesis. These more severe manifestations are associated with pronounced ataxia, severe immune dysfunction, and significantly reduced life expectancy. Splicing mutations and insertions also impair ATM function, though the degree of disruption varies depending on the mutation’s genomic location. Missense mutations, which involve amino acid substitutions that do not prevent ATM activity entirely, are linked to milder disease courses, with later onset of ataxia and less-pronounced immune deficiency.

These findings support the conclusion that ATM mutation type is a key predictor of Louis-Bar syndrome severity. Nevertheless, even patients with identical mutations show considerable variability in their clinical presentation, suggesting the involvement of additional modifying factors [21]. Polymorphisms in genes regulating the cell cycle and apoptosis may modulate disease progression, either attenuating or exacerbating symptoms [22]. Epigenetic mechanisms, such as promoter methylation of the ATM gene, may also play a crucial role in phenotypic variability by influencing gene expression levels [23].

The age at symptom onset is another critical factor affecting disease severity. In cases with mutations that greatly disrupt ATM protein synthesis, ataxia and immune dysfunction manifest in early childhood, while partially functional protein variants result in a more gradual disease progression [24]. Phenotypic variability is also observed in heterozygous carriers of ATM mutations, who face an elevated risk of malignancies, further underscoring ATM’s significance in cancer pathogenesis [25].

Data analysis confirms that the severity of Louis-Bar syndrome is determined not solely by the type of ATM mutation, but also by a combination of genetic and epigenetic factors. Identifying these modifying influences is crucial for personalised diagnostic approaches and developing novel therapeutic strategies aimed at decelerating disease progression.

### Diagnosis and contemporary approaches to treatment

Diagnosis of A-T poses a considerable clinical challenge due to the broad spectrum of manifestations and the necessity for differential diagnostic approaches. Initial symptoms, such as progressive cerebellar ataxia, oculomotor apraxia, and telangiectasias, may appear at varying ages, which complicates early recognition of the disease. Neurological, immunological, and oncological manifestations also play a crucial role in the diagnostic process, given the high incidence of malignancies and immunodeficiency states associated with the condition [10].

Anamnesis collection is a key component of diagnosis, as it enables clinicians to determine the age of disease onset, symptom progression, and potential risk factors. Particular emphasis is placed on family history (including relatives with similar symptoms), frequency of infectious diseases, and specific features of early childhood development [13].

Neurological assessment is used identify cerebellar ataxia, dysarthria, oculomotor apraxia, and other motor and coordination disorders associated with the disease. Evaluation includes muscle strength and tone; reflexes; Romberg’s posture stability; and the ability to perform precise movements. The severity of ataxia is graded using GMFCS, MACS, and CFCS scales, providing a clinical picture of motor and communication impairments [3].

For an in-depth assessment of nervous system involvement in A-T, electrophysiological methods are employed, including electroneuromyography (ENMG) and evoked potential studies (visual evoked potentials, or VEPs; somatosensory evoked potentials, or SSEPs; and motor evoked potentials, or MEPs). ENMG enables the detection of peripheral neuropathy, which is characterized by decreased conduction velocity and signs of axonal degeneration, indicating impaired peripheral nerve function. VEPs are used to detect subclinical lesions of the visual pathways and assess signal transmission from the retina to the visual cortex. SSEPs assist in diagnosing somatosensory pathway disorders, particularly within the spinothalamic tract, that may potentially present as altered sensory perception. MEPs allow assessment of motor neuron dysfunction and central motor conduction abnormalities. These techniques are critical for differential diagnosis, as the clinical picture of ataxia in A-T may resemble spinocerebellar degenerations, hereditary polyneuropathies, and other neurodegenerative diseases [14].

Magnetic resonance imaging (MRI) of the brain is a pivotal tool for detecting neurodegenerative changes. In patients with A-T, progressive cerebellar atrophy is commonly observed. Characteristic MRI findings include reduction in the volume of the cerebellar vermis, enlargement of the posterior fossa cistern, and cortical thinning of cerebellar gyri [32].

Laboratory investigations include measurement of immunoglobulin levels (IgA, IgG) and alpha-fetoprotein (AFP) using nephelometry. A typical laboratory profile in A-T includes decreased IgA and IgG levels, as well as elevated AFP, which reflect a combination of immunodeficiency and impaired DNA repair mechanisms. Polymerase chain reaction (PCR) diagnostics for viral and bacterial pathogens are also conducted, as A-T patients are predisposed to frequent infections, including Epstein-Barr virus, cytomegalovirus, and herpes virus infections [17].

The γH2AX assay is employed to evaluate DNA instability by detecting double-strand breaks. This method reveals increased cellular sensitivity to DNA damage, serving as a molecular marker of A-T and providing insights into the pathogenesis of the disorder [19].

Assessment of pulmonary function is essential due to the high prevalence of bronchial obstruction, bronchiectasis, and respiratory insufficiency in A-T. Spirometry is used to evaluate vital capacity and bronchial patency, while pulse oximetry and blood gas analysis help identify chronic hypoxemia and respiratory failure. These tests are particularly relevant in advanced stages of the disease, when immunodeficiency and recurrent infections contribute to declining pulmonary function [23].

Molecular genetic diagnostics remain the gold standard for confirming the diagnosis. Next-generation sequencing allows the identification of point mutations in the ATM gene, while multiplex ligation-dependent probe amplification (MLPA) detects large deletions and duplications. Genetic testing is especially critical in populations with a high prevalence of consanguinity, where the probability of homozygous ATM mutations is significantly increased [12, 19].

Pedigree analysis is conducted to identify hereditary risk factors and assess the degree of parental consanguinity, which is of particular significance in autosomal recessive disorders such as A-T. In cases of consanguineous parentage, the likelihood of inheriting identical mutated ATM alleles is significantly elevated, increasing the risk of the disease in offspring [25].

Table 1 presents key diagnostic methods employed in the identification of ataxia-telangiectasia.

**Table 1.** Diagnostic methods for ataxia-telangiectasia (Louis-Bar Syndrome).

**Method**	**Purpose**
Clinical examination	Detection of ataxia, nystagmus, telangiectasia, dysarthria
Electrophysiology	ENMG (electroneuromyography) – diagnosis of peripheral neuropathy; evoked potentials – assessment of sensory impairments
Brain MRI	Identification of cerebellar atrophy, enlargement of the cisterna magna
Laboratory tests	IgA, IgG, AFP (nephelometry), PCR for infections, γH2AX assay – evaluation of DNA instability
Pulmonary function tests	Spirometry, pulse oximetry, blood gas analysis – diagnosis of broncho-obstructive syndrome
Genetic testing	NGS, MLPA – confirmation of ATM, gene mutations, pedigree analysis

*Source:* compiled based on data analysis [3, 12, 14, 17,18,19, 23, 24, 32].

The diagnostic approaches outlined demonstrate the necessity of a comprehensive strategy. Clinical signs of ataxia raise initial suspicion, but require confirmation through laboratory and instrumental testing. Electrophysiological studies refine the evaluation of neurological involvement, while brain MRIs reveal structural changes typical of A-T. Laboratory assays are crucial for assessing immunodeficiency, and γH2AX assays offer insight into impaired DNA repair mechanisms. Pulmonary function tests help detect respiratory insufficiency in later stages of the disease, which is vital for determining a prognosis. Molecular genetic testing remains the most accurate method for confirming the diagnosis and determining the patient’s mutation status.

As A-T is an incurable condition, treatment aims to slow symptom progression, improve quality of life, and prevent complications. Supportive therapy includes the administration of L-carnitine, coenzyme Q10, and B-group vitamins, which enhance neuronal energy metabolism and support mitochondrial function [10]. Alpha-lipoic acid and vitamin E may also be used as antioxidants to protect cells from oxidative stress – a significant factor in the disease’s pathogenesis [18].

Immunodeficiency correction is achieved through immunoglobulin replacement therapy (intravenous IgG), which reduces the frequency of respiratory infections – one of the leading causes of mortality in A-T patients [17]. Management of infectious complications involves the use of mucolytics, macrolides, and inhaled antibiotics, which enhance bronchial clearance and prevent chronic infection. In cases of persistent inflammation, prolonged antibiotic therapy (guided by pathogen susceptibility) may be indicated [19].

Physiotherapy and respiratory therapy include bronchial drainage, vibratory massage, and specific breathing exercises aimed at improving lung function and preventing bronchiectasis. For patients with impaired cough reflex, the use of insufflator-exsufflator devices and positive expiratory pressure (PEP) systems is recommended to reduce the risk of respiratory complications [23].

Genetic counselling is a critical element of preventive care. In families with a high risk of A-T, prenatal diagnostics (PCR testing of amniocytes) or preimplantation genetic testing (PGT-M) are advised during assisted reproductive procedures [26] (Table 2).

**Table 2.** Treatment approaches for ataxia-telangiectasia (Louis-Bar Syndrome).

**Therapeutic approach**	**Treatment methods**	**Expected outcome**
Supportive therapy	L-carnitine, coenzyme Q10, B vitamins, alpha-lipoic acid, vitamin E	Improvement of neuronal energy metabolism, reduction of oxidative stress
Immunomodulation	Intravenous immunoglobulin (IVIG) therapy	Reduced frequency of infectious complications, maintenance of immune function
Antibacterial therapy	Mucolytics, macrolides, inhaled antibiotics, prolonged antibiotic courses for chronic infection	Improved bronchial patency, prevention of chronic respiratory infections
Respiratory therapy	Bronchial drainage, vibratory massage, breathing exercises, mechanical insufflation-exsufflation devices, positive expiratory pressure (PEP) therapy	Enhanced pulmonary function, prevention of bronchiectasis and respiratory failure
Physiotherapy and rehabilitation	Targeted exercises, coordination training, stabilisation techniques	Slowed progression of ataxia, maintenance of motor function
Genetic counselling	Prenatal diagnosis (PCR analysis of amniocytes), preimplantation genetic testing for monogenic disorders (PGT-M)	Reduced risk of offspring with ataxia-telangiectasia

*Source:* compiled based on data analysis [10, 17,18,19, 23, 26].

A comprehensive therapeutic approach includes supportive care, immunocorrection, respiratory therapy, physiotherapy, and genetic counselling. Supportive care aims to improve metabolic processes in neural tissue and reduce oxidative stress (a significant contributor to the progression of degenerative changes). Immunocorrection helps decrease the frequency of infections, while respiratory therapy prevents complications in the pulmonary system, which represent one of the leading causes of mortality. Physiotherapeutic methods play a crucial role in maintaining patients’ motor activity. Specialised exercises and rehabilitation programmes allow for the slowing of ataxia progression, while the use of respiratory trainers and vibratory therapy alleviates bronchial obstruction. Genetic counselling is particularly important for families with a history of A-T. The use of genetic screening, preimplantation diagnostics, and prenatal testing allows for the prevention of births affected by this condition.

Palliative care in A-T focuses on improving patients’ quality of life, slowing the progression of neurological and respiratory complications, and minimising discomfort. A key aspect of palliative care is respiratory support including bronchial drainage, vibratory massage, and positive expiratory pressure (PEP) devices to prevent pulmonary infections and bronchiectasis. For patients with pronounced respiratory impairment, non-invasive ventilation (NIV) and oxygen therapy may be indicated. Management of swallowing difficulties requires dietary support, food texture modification, and, if necessary, gastrostomy placement. Muscle relaxants and analgesics are employed in cases of severe neurological disorders accompanied by spasticity and pain. In addition, psychological and social support for patients and their families is essential for facilitating adaptation to the chronic nature of the disease; providing education in caregiving techniques; and promoting conditions for maximal patient autonomy [10].

Diagnosis of A-T requires a combination of clinical, laboratory, instrumental, and molecular-genetic methods for timely identification of the disease and assessment of its severity. Treatment is aimed at maintaining bodily functions and improving patients’ quality of life. Immunocorrection, respiratory therapy, physiotherapy, and genetic counselling constitute the foundation of the current management strategy for individuals with ataxia-telangiectasia.

### Clinical case: observation of a family with three affected children

This study presents a clinical observation of a family in which three children were diagnosed with A-T, with confirmation by molecular genetic testing. This case illustrates the core manifestations of the disease and emphasises the importance of comprehensive diagnostics and supportive care.

The eldest child (Patient 1) exhibited the most severe clinical presentation, with signs of ataxia manifesting at the age of three as clumsiness, difficulty performing precise movements, and gait disturbances. He developed additional symptoms by the age of five, including scanning speech, intention tremor, and episodes of dysphagia. His progressive neurological deficit led to pronounced dysarthria, limited independence in daily activities, and complete dependence on external assistance. Due to Patient 1’s ataxic features, his condition could be mistakenly interpreted as ataxic cerebral palsy; he displayed genetically determined neurodegeneration of the cerebellum, however, which is not seen in cerebral palsy caused by perinatal hypoxia or intrauterine infection. This underscores the importance of thorough differential diagnosis.

Neurological status was assessed using international scales. Level III of the GMFCS indicated that the child could ambulate only with assistance – in this case, using a crutch or parental support – and lacked self-care ability, requiring help with feeding, dressing, and other daily tasks. His CFCS score was II, reflecting his preserved cognitive function but marked speech impairment – notably, his scanning speech.

**Figure 2.**
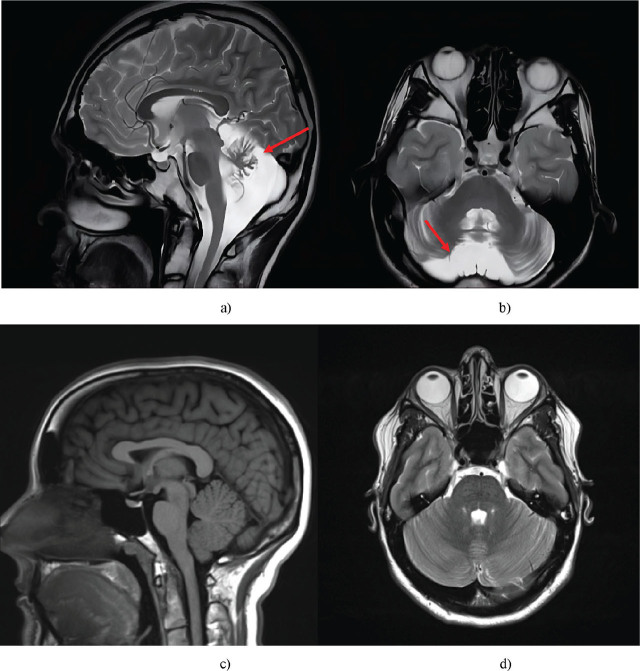
Brain MRI of the eldest child (Patient 1): a) sagittal; b) axial sections. Healthy brain: c) sagittal; d) axial slices. The pathological features observed include severe cerebellar vermian atrophy, compensatory enlargement of the posterior cerebellar cistern, and marked decrease in overall cerebellar volume, with thinning of the cerebellar folia and deepening of the cerebellar sulci. *Source*: a-b were obtained during the study and c-d are reproduced from [34].

A brain MRI of Patient 1 revealed degenerative changes characteristic of ataxia-telangiectasia. The sagittal slice demonstrated significant atrophy in the cerebellar vermis, with compensatory expansion of the posterior cerebellar cistern. Axial slices confirmed decreased cerebellar volume, thinning of the gyri, and deepening of the sulci – all indicative of pronounced atrophic processes (Figure 2).

MRI findings correlated with the clinical picture included severe cerebellar ataxia, dysarthria, dysphagia, and nystagmus. Functional assessment according to the GMFCS, MACS, and CFCS scales indicated substantial motor impairment and the need for continuous support. Despite preserved intelligence, limited mobility and difficulties with self-care rendered the patient dependent on others.

The middle child (Patient 2) exhibited less-severe neurological deficits compared to the elder brother. The first signs of ataxia appeared at the age of four, initially presenting as intermittent gait instability, particularly on uneven surfaces. Subsequently, Patient 2’s coordination difficulties gradually increased, manifesting as episodes of dysmetria during purposeful movements and mild intention tremor. In contrast to the elder child, Patient 2 demonstrated only minimal dysarthria, with speech remaining intelligible – though slight articulation inaccuracy was noted, particularly during fatigue. Neurological examination revealed moderate cerebellar ataxia, evident in slowed and mildly uncoordinated movements, as well as mild limb hypotonia. Sensory function and cognitive development were age-appropriate, distinguishing Patient 2’s clinical course from the more significant motor impairments observed in the older sibling.

**Figure 3.**
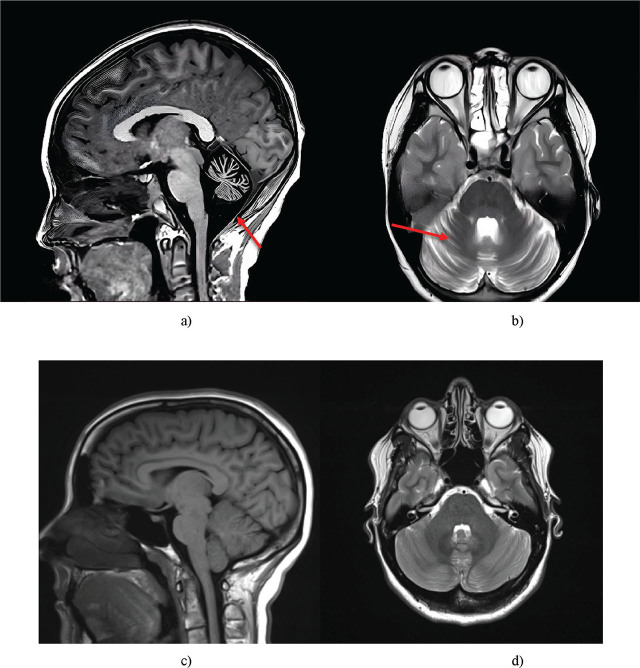
Brain MRI of the middle child (Patient 2): a) sagittal; b) axial sections*. Healthy brain: c) sagittal; d) axial slices. The pathological features observed include moderate vermian thinning and cerebellar hemispheric subatrophy with moderate sulcal widening. *Source:* a-b were obtained during the study and c-d are reproduced from [34].

Patient 2’s brain MRI revealed moderate cerebellar atrophy. The sagittal slice showed thinning of the cerebellar vermis, suggesting progressive neurodegeneration, although to a lesser extent than in the elder brother. Axial slices demonstrated subatrophy of the cerebellar hemispheres, with moderate sulcal deepening; however, the severity of these changes was significantly less than in Patient 1. The visualised neurodegenerative processes was in agreement with the clinical data, confirming a milder disease course in Patient 2 compared to the elder sibling (Figure 3).

Based on Patient 2’s MRI data and clinical examination, it can be assumed that Patient 2’s degree of cerebellar degeneration is directly correlated with the severity of ataxia and neurological symptoms. Unlike the older brother, who exhibited pronounced cerebellar atrophy accompanied by compensatory enlargement of the posterior cerebellar cistern, Patient 2’s changes were milder, explaining the less severe disease course. This underscores the importance of early detection of structural changes for predicting disease progression and development of individualised rehabilitation strategies.

The youngest child (Patient 3) presented with the mildest disease course among the brothers. At the age of 5, he exhibited only minor coordination disturbances, manifested as occasional gait instability. Episodic tremor was minimally expressed and did not significantly impact daily functioning. Unlike his older siblings, his speech function remained largely intact, with no evidence of dysarthria. His ataxia was subclinical in nature.

**Figure 4.**
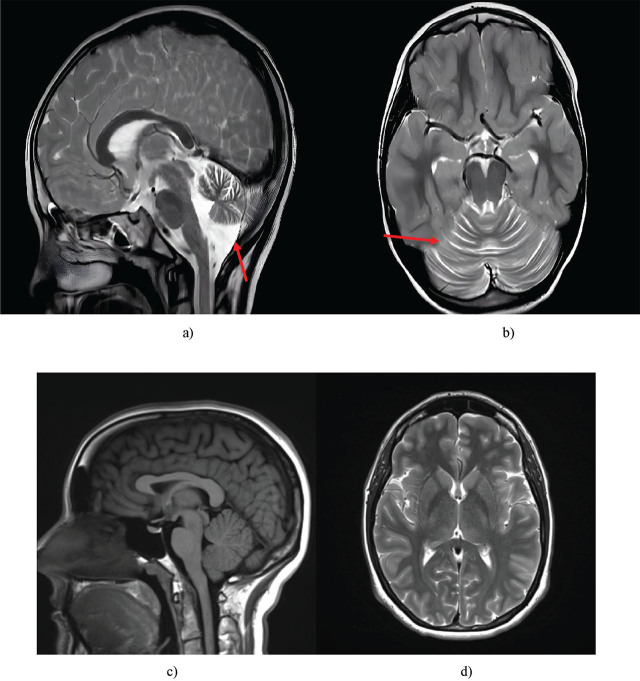
Brain MRI of the youngest child (Patient 3): a) sagittal; b) axial sections*. Healthy brain: c) sagittal; d) axial slices. The pathological features observed include minimal vermian thinning and subtle sulcal widening with relatively preserved cerebellar volume. *Source:* a-b were obtained during the study and c-d are reproduced from [34].

MRI findings confirmed the presence of cerebellar subatrophy; however, the extent of changes was considerably less pronounced than in his older siblings. The sagittal section revealed slight thinning of the cerebellar vermis, indicative of an early-stage degenerative process. The axial image demonstrated subtle sulcal deepening, minimal reduction in cerebellar volume, and no distinct signs of the severe atrophic changes characteristic of more advanced forms of A-T (Figure 4). These findings are in line with Patient 3’s relatively mild clinical presentation, confirming the marked phenotypic variability of A-T, even within a single family.

The detected differences confirm the intra-familial variability in the disease’s course, even among closely related individuals with the same mutation of the ATM gene. Patient 3’s degenerative cerebellar changes were less pronounced than in his older siblings, which may reflect the influence of additional factors, such as individual compensatory mechanisms or epigenetic modifications.

**Figure 5.**
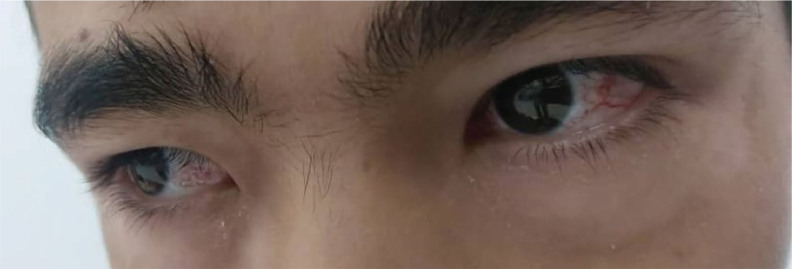
Telangiectasias on the conjunctivae of the eldest child, appearing as “spider-like” vascular lesions. *Source:* obtained during the course of the study.

Telangiectasias were identified in two of the three children, but their severity varied significantly. In the eldest child, telangiectasias were most prominent and located on the conjunctivae of the eyes, forming the characteristic vascular “spider” patterns (Figure 5).

This manifestation is typical for A-T and reflects systemic microcirculatory disturbances associated with DNA-repair defects. In the middle child, telangiectasias were present but much less prominent, which may indicate a milder disease course. In the youngest child, telangiectasias were absent, further demonstrating the condition’s significant phenotypic variability, even among patients with identical genetic mutations.

**Figure 6.**
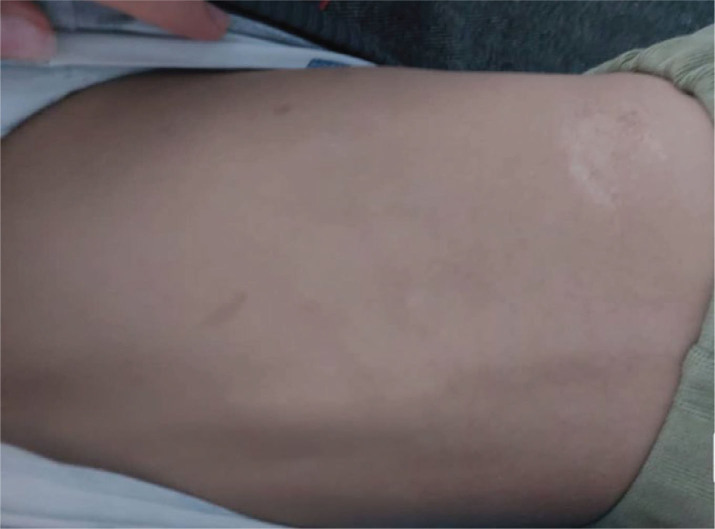
Skin lesions in the youngest child: “café-au-lait” spots and an area of skin induration. *Source:* obtained during the course of the study.

In addition to vascular abnormalities, the youngest child exhibited additional cutaneous manifestations not observed in his older brothers. Hyperpigmented “café-au-lait” macules and areas of skin induration were identified on the patient’s back, suggesting a possible co-occurrence of A-T with other genetic factors influencing phenotype (Figure 6).

These skin changes are relatively subtle, but warrant further investigation, including potential involvement of other genes responsible for the regulation of cell growth and differentiation processes.

Laboratory findings in all three children confirmed the presence of immunodeficiency, a key diagnostic feature of ataxia-telangiectasia. Reduced levels of immunoglobulins IgA and IgG indicated humoral immune dysfunction, which accounted for the patients’ predisposition to recurrent respiratory infections. Elevated levels of alpha-fetoprotein, detected in all three children, served as an additional biochemical marker, confirming impaired cellular maturation and regeneration processes.

**Figure 7.**
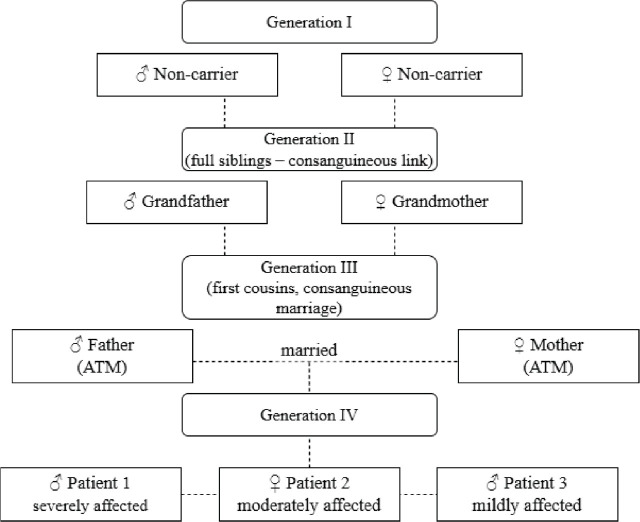
Family pedigree, demonstrating consanguineous marriage in the third generation. *Source:* based on data from the present study.

Genetic testing validated the patients’ diagnosis and identified the specific mutation responsible for the disease. Next-generation sequencing revealed a homozygous mutation in the ATM gene (NM_000051.3:c.5932G>A; NP_000042.3:p.Asp1978Glu), resulting in the substitution of aspartic acid with glutamic acid at position 1978 of the protein. This mutation has previously been described in the scientific literature as pathogenic, and it is associated with the classical form of A-T. Pedigree analysis revealed consanguinity in the third generation, with the maternal grandmother and paternal grandfather being full siblings (Figure 7). This consanguineous relationship increased the likelihood of homozygous inheritance of the pathogenic mutation, thereby highlighting the role of inbreeding in elevating disease risk.

A comparative analysis of these three clinical cases revealed marked phenotypic variability in the disease. The most severe manifestation was observed in the eldest child, who was diagnosed with pronounced cerebellar ataxia, accompanied by severe coordination deficits, scanning speech, and significant motor impairment. The middle child exhibited a milder disease course, with moderate ataxic symptoms and minimal speech disturbances. The youngest child presented with only minor coordination issues and minimal neurological deficits.

Differences in the patients’ clinical presentation correlated with neuroimaging findings. The eldest child exhibited marked cerebellar atrophy with reduced vermis volume and deep cerebellar sulci on axial MRI scans, consistent with his severe clinical course. The middle child demonstrated less-pronounced subatrophic changes in the cerebellum, corresponding to the milder neurological manifestations. In the youngest child, MRI revealed only minimal cerebellar atrophy, confirming his relatively benign disease progression.

## Discussion

A valid interpretation of the results requires correlation of the obtained clinical and laboratory data with existing literature on A-T. A critical aspect is the evaluation of phenotypic variability, which facilitates identification of factors influencing the severity of neurological, immunological, and genetic manifestations.

Molecular genetic analysis of the patients confirmed a homozygous mutation in the ATM gene (NM_000051.3:c.5932G>A; NP_000042.3:p.Asp1978Glu), associated with the classical form of A-T. This mutation results in ATM protein dysfunction, impairing DNA repair mechanisms and increasing cellular radiosensitivity. A study by Shiloh and Ziv [12] demonstrated that ATM dysfunction disrupts cell cycle control and leads to the accumulation of DNA damage, thereby increasing the risk of malignancies. Similar findings were reported by Bensimon et al. [13], who investigated ATM’s role in intracellular signalling under stress conditions. Furthermore, Cremona and Behrens [14] confirmed the link between ATM mutations and oncogenesis, explaining the high incidence of neoplastic diseases in patients with ataxia-telangiectasia.

The identified ATM gene mutation impaired the repair of DNA double-strand breaks, leading to heightened genomic instability and systemic dysfunction. Neuroimaging data confirmed significant cerebellar atrophy in the parents that was correlated with the severity of their clinical symptoms. Research by Goodarzi et al. [15] demonstrated that ATM-dependent DNA repair pathways are activated in response to chromatin damage to maintain genomic integrity. Additionally, Lavin and Shiloh [16] confirmed that ATM dysfunction not only contributes to genomic instability, but also results in profound immunological disturbances, explaining the immunodeficiency observed in patients in the present study.

Immunological studies confirmed a marked immunodeficiency in all patients, evidenced by decreased IgA and IgG levels and elevated alpha-fetoprotein. These findings indicate the disrupted B-cell maturation and genomic instability that are characteristic of A-T. A correlation was identified between the degree of immunodeficiency and the frequency of infectious complications; the most pronounced alterations were found in the eldest child, who had a history of recurrent pneumonia and bronchitis. Van Os et al. [17] reported that immunodeficiency in patients with this condition involves not only antibody deficiencies, but also defects in DNA repair mechanisms, which increase cellular vulnerability and the risk of malignancies. Watters [18] emphasised the critical role of oxidative stress in the disease’s pathogenesis, which may explain the patients’ heightened susceptibility to viral and bacterial infections. Furthermore, studies by Stankovic et al. [22] demonstrated that ATM dysfunction reduces lymphocyte proliferation, exacerbating immunodeficiency and elevating the risk of lymphoproliferative disorders.

Each of the patients’ brain MRIs revealed significant cerebellar atrophy, with its severity correlating with clinical manifestations. All examined children showed reduced cerebellar volume, thinning of the gyri, and deepened sulci, with the most pronounced changes in the eldest child, consistent with the most severe disease course. It has been established that cerebellar degeneration is a key morphological hallmark of ataxia-telangiectasia, as supported by data from Jacquemin et al. [21], who demonstrated reduced cerebellar volume in patients with various ATM mutations. The study by Concannon and Gatti [19] revealed substantial diversity in ATM gene mutations, which were associated with variable degrees of cerebellar atrophy and neurological symptoms. Telatar et al. [20] also confirmed the phenotypic heterogeneity of the disorder by highlighting differences in brain morphological changes among patients with different ATM mutations.

Clinical manifestations in the examined patients exhibited considerable variability despite the presence of an identical mutation in the ATM gene. Differences in the rate of disease progression and the degree of cerebellar atrophy were identified, indicating the influence of additional factors, such as epigenetic mechanisms and individual the immune-response characteristics. Rawat et al. [23] also found marked clinical heterogeneity among patients with A-T, reporting cases with atypical presentation, later onset of symptoms, and milder ataxia. Woods et al. [24] emphasised that clinical manifestations may vary even among patients with identical mutations, and attributed this to the influence of other genes, as well as environmental influences. Veenhuis et al. [28] noted that phenotypic variability may be associated with differing levels of residual ATM protein, which may explain the patients’ differences in the severity of ataxia and immunodeficiency.

Pedigree analysis revealed a consanguineous marriage in the third generation, supporting the role of inbreeding in increasing the risk of homozygous mutation transmission. The findings suggest that the hereditary transmission of A-T in this family occurred due to a high degree of parental relatedness, in line with other population-based studies. Research by Bittles [25] confirms that the prevalence of autosomal recessive disorders, including A-T, is elevated in populations with high consanguinity rates. Bennett et al. [26] stress that consanguineous unions significantly raise the risk of inheriting rare pathogenic mutations, underscoring the importance of genetic counselling as a preventive strategy. Bittles and Black [29] further highlight that inbreeding not only increases the likelihood of recessive disorders but may also exacerbate clinical severity, a phenomenon also observed in this family.

This study confirms substantial clinical heterogeneity in A-T, even among patients harbouring the same ATM mutation. Identified differences in the severity of neurological symptoms, the extent of cerebellar atrophy, and the degree of immunodeficiency all underscore the need for a personalised approach to diagnosis and treatment.

The findings are consistent with previous research highlighting the impact of genetic and epigenetic factors on disease progression. The combined use of molecular-genetic testing, MRI, and functional assessments enables more precise determination of neurological involvement and facilitates disease prognosis. The integration of prenatal and postnatal diagnostics in regions with a high frequency of consanguinity may help reduce the prevalence of A-T and improve patients’ quality of life.

Modern neuroimaging techniques provide diagnostic capabilities for A-T that allow for detection of characteristic changes long before the onset of overt clinical symptoms. MRI findings in the patients confirmed cerebellar atrophy of varying degrees. Comparable results are presented by Sahama et al. [32], who report that neurodegenerative changes can be visualised at early disease stages to facilitate timely diagnosis. The findings of Cavalli-Sforza and Bodmer [30] underscore the significance of an integrated approach to diagnosing hereditary diseases, incorporating clinical, molecular-genetic, and radiological evaluations. Modell and Darr [31] advocate for the inclusion of routine MRI diagnostics in the assessment protocol for suspected A-T cases, as early visualisation of cerebellar changes enables disease trajectory prediction and therapeutic optimisation.

Early clinical manifestations of A-T may mimic the ataxic subtype of cerebral palsy (CP), complicating differential diagnosis. According to UpToDate [35], both conditions are characterised by cerebellar ataxia, dysmetria, intention tremor, and balance impairment; A-T, however, shows progressive symptomatology, in contrast to the static nature of CP. Additional features of A-T that are not typically observed in CP include immunodeficiency, elevated alpha-fetoprotein (AFP), and telangiectasias. MRI serves as a key tool for differentiation: A-T is associated with progressive cerebellar atrophy, whereas CP often reveals congenital anomalies.

Therapeutic strategies in the management of A-T have focused primarily on maintaining vital bodily functions, slowing symptom progression, and preventing complications. Particular emphasis has been placed on metabolic therapy, including the administration of L-carnitine, coenzyme Q10, and B-group vitamins, all of which enhance neuronal energy metabolism. Additionally, antioxidants such as alpha-lipoic acid and vitamin E are employed to reduce oxidative stress, which is one of the key factors in the neurodegeneration associated with this disorder.

The disease’s associated immune dysfunction necessitated immunoglobulin replacement therapy, with the intent of compensating for IgG deficiency and reducing the incidence of infectious complications. Control of infections has been achieved through the use of inhaled antibiotics, macrolides, mucolytics, and bronchodilators, particularly in children with obstructive pulmonary changes. Patients required ongoing respiratory therapy, including bronchial drainage, vibratory chest physiotherapy, the use of positive expiratory pressure (PEP) devices, and insufflation-exsufflation systems, to facilitate bronchial secretion clearance.

Genetic counselling has become an integral component of family management due to the high risk of recurrence, with offspring having the same pathology. Parents were advised to consider preimplantation genetic testing for monogenic diseases (PGT-M) in future planned pregnancies to prevent transmission of the pathogenic mutations.

This case series vividly illustrates that even within a single family sharing an identical ATM gene mutation, the severity of A-T can vary significantly. The phenotypic variability of A-T underscores the necessity of a comprehensive diagnostic approach incorporating molecular genetic testing, neuroimaging, and laboratory investigations. Individualised treatment regimens targeting immune deficiency correction, neuroprotection, and respiratory support will play a pivotal role in improving patients’ quality of life. Early diagnosis and timely medical intervention are crucial for decelerating disease progression and minimising complications.

## Conclusions

A comprehensive analysis of the clinical, immunological, and neuroimaging characteristics of A-T was conducted in three siblings with a confirmed ATM gene mutation. A correlation was established between the severity of cerebellar atrophy and the intensity of neurological symptoms. The eldest child presented with the most pronounced ataxia, dysarthria, and impaired coordination and motor function, accompanied by significant cerebellar atrophy. The middle child exhibited milder neurological deficits, while the youngest displayed minimal symptoms, which was corroborated by MRI findings demonstrating less marked atrophic changes.

Molecular-genetic testing confirmed a homozygous ATM variant (NM_000051.3:c.5932G>A; NP_000042.3:p. Asp1978Glu), associated with the classical form of ataxia-telangiectasia. Pedigree analysis revealed a consanguineous marriage in the third generation, increasing the risk of homozygous mutation inheritance. This finding highlights the need for genetic counselling in families with a high probability of autosomal recessive conditions. The study also confirmed substantial phenotypic variability even within a single family. Differences in the severity of neurological and immunological symptoms were dependent on the degree of cerebellar atrophy and immunodeficiency, reinforcing the need for an individualised approach to patient management.

An analysis of diagnostic strategies demonstrated that next-generation sequencing is the most accurate and informative method for identifying pathogenic ATM mutations. Brain MRI identified key morphological disease markers, while electrophysiological studies (evoked potentials and electromyography) helped delineate central and peripheral nervous system involvement. Immunological assays confirmed marked immunodeficiency, the severity of which correlated with the clinical course. The most severe immunodeficiency was observed in the eldest child, who also experienced frequent and recurrent respiratory-tract infections.

A comparative analysis of therapeutic approaches revealed that immunoglobulin replacement therapy reduces the frequency of infectious complications, but does not affect the progression of neurological symptoms. The use of antioxidants (L-carnitine, coenzyme Q10, and vitamin E) provided moderate benefits in maintaining cognitive and motor functions. Physiotherapeutic interventions, respiratory support, symptomatic treatment, and social integration are essential components of care for patients with progressive forms of A-T.

The findings underscore the importance of a multidisciplinary approach to managing A-T. An optimal strategy involves regular immunomodulation, control of infectious complications, early initiation of symptomatic therapy, and genetic counselling for families.

This study is limited by the small number of participants, attributable to the rarity of ataxia-telangiectasia. The analysis was based on a single family, limiting its generalisability to broader populations. The absence of long-term follow-up data precludes assessment of the sustained efficacy of therapeutic strategies. Future directions include expanding research to larger cohorts with diverse ATM genotypes to better elucidate factors influencing phenotypic variability. Investigations into the efficacy of novel therapeutic strategies, including targeted mutation correction, are warranted. Longitudinal studies will aid in refining prognostic models and developing more effective approaches to supportive care.
